# Effectiveness of diabetes self-management education interventions on glycemic control in persons with diabetes in Africa: a systematic review and meta-analysis

**DOI:** 10.1097/MS9.0000000000003420

**Published:** 2025-05-30

**Authors:** Eric Peprah Osei, Emmanuel Ekpor, Samuel Akyirem, Amos Asante, Debby Syahru Romadlon

**Affiliations:** a College of Nursing, University of Illinois Chicago, Chicago, IL, USA; b School of Nursing and Midwifery, University of Ghana, Accra, Ghana; c Yale School of Nursing, Yale University, New Haven, Connecticut, USA; d Ghana Health Service, Atebubu Government Hospital, Atebubu, Ghana; e Faculty of Nursing, Chulalongkorn University, Bangkok, Thailand

**Keywords:** Africa, diabetes, glycemic control, self-management education, systematic review

## Abstract

**Background:**

Achieving effective glycemic control remains a major challenge in Africa, with over 50% of individuals with diabetes not meeting recommended targets. This systematic review aimed to evaluate the effectiveness of diabetes self-management education (DSME) interventions on glycemic control (HbA1c) in persons with diabetes in Africa.

**Methods:**

Studies were retrieved from searches conducted on PubMed, CINAHL, Scopus, Web of Science, and Cochrane Library, from their inception to 9 June 2024. The search strategy included keywords and database-specific indexed terms such as (i) diabetes, (ii) diabetes self-management education, (iii) glycemic control, and (iv) Africa. The DerSimonian-Laird random effect model was used to pool the mean difference in HbA1c levels. Heterogeneity across studies was assessed using the *I*^2^ statistic, and subgroup analyses were conducted to explore sources of variability.

**Results:**

Seventeen randomized controlled trials involving 3730 participants met the inclusion criteria. Most DSME interventions were delivered in person, with only two studies utilizing mobile health (mHealth) approaches. The meta-analysis showed a significant reduction in HbA1c levels in the DSME intervention compared to usual care, with a pooled mean difference of −1.02% (95% CI −1.46 to −0.58). Subgroup analyses revealed variations in effectiveness based on intervention characteristics; however, these were not statistically significant.

**Conclusion:**

DSME interventions significantly improve glycemic control compared to usual care among people with diabetes in Africa. Future research should investigate context-specific factors that may influence the effectiveness of these interventions.

## Introduction

Diabetes has emerged as a significant global health challenge, with its prevalence increasing dramatically over recent decades. The Global Burden of Disease (GBD) study reports a 90.5% rise in the age-standardized prevalence of diabetes over the past 30 years^[1]^. Projections indicate this upward trend will continue, particularly in low- and middle-income countries (LMICs). This is especially concerning in Africa, where the number of adults living with diabetes is expected to increase from approximately 24 million in 2021 to 55 million by 2045^[2]^. Moreover, Africa bears the highest age-standardized diabetes-related mortality rate globally^[1]^, a disparity largely attributed to the continent’s limited capacity for diabetes management^[3]^.

**HIGHLIGHTS**

- Meta-analysis revealed that DSME interventions significantly improve glycemic control among individuals with diabetes in Africa.
- Effectiveness of DSME interventions varied across individual studies, highlighting the need for tailored approaches.
- Identifying contextual factors that influence DSME success in diverse African settings is essential for optimizing outcomes.
- Digital health technologies for DSME remain largely unexplored in African contexts, presenting a key area for future research.

Achieving effective glycemic control is a key challenge in diabetes management across Africa. Globally, about 50% of individuals with diabetes reach target glycemic levels, defined as hemoglobin A1c (HbA1c) levels of 7% or lower^[4]^. However, this proportion is considerably lower in African countries. For instance, only 33.2% of individuals in an Ethiopian study met the target^[5]^, and in Uganda, the proportion was just 15.7%^[6]^. Consolidated evidence across 16 sub-Saharan African countries revealed that only 30.3% (95% CI: 27.6–32.9) of glycemic control targets were met^[7]^. This findings highlight an urgent need for more effective diabetes management strategies tailored to the African context.

Diabetes self-management education (DSME) is one such promising strategy. DSME is a structured, evidence-based educational intervention aimed at building skills, knowledge, and confidence in self-care. It empowers individuals with diabetes to make informed decisions, adopt healthy behaviors, and manage their condition effectively^[8,9]^. DSME interventions have been shown to improve a variety of clinical outcomes, including glycemic control^[10]^, and are associated with reduced risks of complications, hospitalization, and all-cause mortality^[11,12]^. They also enhance behavioral outcomes by fostering self-efficacy, promoting active patient engagement, and developing problem-solving abilities essential for long-term diabetes management^[8]^.

In addition to being effective, DSME interventions are considered cost-efficient – an important advantage in resource-constrained settings such as many parts of Africa^[13]^. However, several barriers may limit their implementation and effectiveness. Many individuals face prohibitive costs related to transportation to healthcare facilities and the expenses associated with diabetes supplies^[14]^. Additionally, the healthcare infrastructure in many parts of Africa faces shortages of trained healthcare professionals, limited diabetes educational resources, and under-resourced facilities^[4]^, all of which may restrict the reach and effectiveness of DSME programs. Cultural beliefs and practices also play a role. For instance, in some communities, traditional beliefs regarding the causes and management of chronic conditions like diabetes may diverge from biomedical approaches^[14]^, posing further challenges to DSME integration.

Despite the documented benefits of DSME interventions, evidence regarding its effectiveness in African settings remains limited. A global systematic review conducted in 2021 included only three studies from Africa when evaluating the effectiveness of DSME on glycemic control^[10]^. A preliminary review of recent literature suggests that additional randomized controlled trials (RCTs) have since been conducted across the continent. However, the evidence remains fragmented, and a comprehensive synthesis is lacking. This gap is particularly concerning given the unique contextual challenges that may influence the implementation and outcomes of DSME interventions in Africa. Against this backdrop, this systematic review aimed to evaluate the effectiveness of DSME interventions on glycemic control among persons with diabetes in Africa, by synthesizing the results of RCTs conducted in the region.

## Methods

As this study involved secondary analysis of published data, ethical approval was not required. The review was conducted in accordance with the Preferred Reporting Items for Systematic Reviews and Meta-Analyses (PRISMA) guidelines^[15]^. The quality of the present study was evaluated using the AMSTAR 2 checklist^[16]^. The protocol for this review was registered in the PROSPERO database.

### Search strategy

Studies were identified from searches conducted on various electronic medical databases, including PubMed, CINAHL, Scopus, Web of Science, and Cochrane Library from their inception to 9 June 2024. No limits were applied to the scope of the search. The search strategy combined keywords and database-specific indexed terms (e.g. MeSH term for PubMed) related to “diabetes,” “diabetes self-management education,” “glycemic control,” and “Africa” (Supplementary Digital Content 1. http://links.lww.com/MS9/A850, Table S1 **Supplementary Digital Content 1:** http://links.lww.com/MS9/A850). The Boolean operators “OR” and “AND” were applied appropriately to optimize the retrieval of relevant studies. Additionally, Africa-specific databases were explored, and hand searching of reference lists of included studies was performed.

### Inclusion and exclusion criteria

The inclusion criteria were studies that 1) involved adults with type 1 or 2 diabetes, 2) were conducted exclusively in any African country, 3) were randomized controlled trials, 4) provided any form of DSME in the intervention arm and usual (standard) care in the control arm, and 5) evaluated the intervention’s effect on glycemic control.

Articles that were ineligible for inclusion consisted of those that included participants with gestational diabetes, were study protocols, had an access-restricted full-text, and were published in non-English language.

### Study selection and data extraction

Duplicate records were initially removed using EndNote, after which the remaining citations were imported into Rayyan for screening. Study selection followed predefined eligibility criteria and was conducted in two stages: first, titles and abstracts were screened; second, full-text articles of potentially eligible studies were reviewed. To reduce the risk of selection bias, two independent reviewers screened all records in parallel.

Data were extracted using a standardized data extraction matrix in Microsoft Excel. The information gathered included authors, year of publication, country, characteristics of participants, characteristics of the DSME intervention, and the outcome measurements for HbA1c. As with the screening process, two independent reviewers conducted the data extraction. Any discrepancies were resolved through discussion, with a third reviewer consulted when consensus could not be reached.

### Quality assessment

The quality of the included studies was assessed by three reviewers using the Cochrane Collaboration Risk of Bias Assessment Tool^[17]^. Discrepancies were resolved by the principal investigator. The quality of studies was evaluated based on seven domains, including selection bias due to random sequence generation or allocation concealment, performance bias, detection bias, attrition bias, reporting bias, and other bias. The risk of bias in each domain was adjudged as high risk, low risk, or some concern in response to a series of “signaling” questions.

### Data analysis

The DerSimonian-Laird random effect meta-analysis was used to pool mean differences (MD) across multiple studies^[18]^. The *metacont* function from the *meta* package in R was used for this meta-analysis^[19]^. Studies that only reported change from baseline HbA1c for intervention and control groups were combined with those that reported post-intervention mean HbA1c for intervention and control groups. MD was calculated using means and standard deviations of post-study HbA1c or change from baseline. For studies that did not report means and standard deviations for HbA1c, the values were derived from available data based on Cochrane guidelines^[18]^. For instance, medians were considered to be approximately equal to means, interquartile ranges were converted to standard deviation by dividing by 1.35, and 95% confidence intervals (CI) were converted to standard deviation using the formula:

$$StandardDeviation=\frac{UpperCI-LowerCI}{2*1.96}*\sqrt{sample}$$

We further conducted subgroup analysis for studies that addressed single vs multiple diabetes self-management domains, had ≤ 6 months vs > 6 months follow-up, delivered intervention in group vs individual format, reported ≥ 20% vs < 20% attrition rates, and originated from North Africa vs sub-Saharan Africa. Publication bias was assessed using Egger’s test and funnel plot^[20]^. The *I*^2^ statistic was used to assess statistical heterogeneity, with values of 25%, 50%, and 75% indicating low, moderate, and high levels of heterogeneity, respectively^[21]^.

## Results

**Figure 1.**
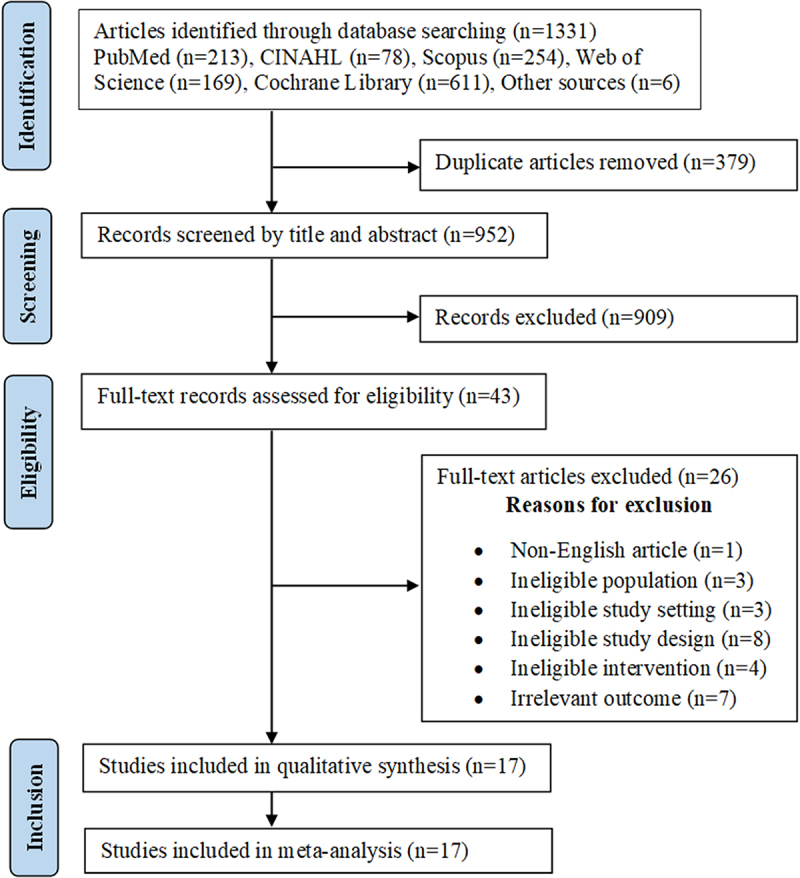
PRISMA flow chart summarizing the article selection process.

A total of 1331 records were identified from our search, of which 379 (28.5%) were duplicates. Title and abstract screening were performed for 952 unique articles, with the full-text of 43 articles assessed for their eligibility. Ultimately, 17 articles were included in this review. Figure 1 presents a summary of the article selection process and the reasons for excluding articles after the full-text review.

### Characteristics of included studies

All the articles included in this review (Table 1) were published after the year 2010, with the majority (64.7%) published since 2018. The trials were conducted in 10 different countries in Africa, spanning both the sub-Saharan and North Africa region. South Africa was the most represented country, with three articles^[22–24]^. This was followed by two articles each from Nigeria^[25,26]^, Ethiopia^[27,28]^, Rwanda^[29,30]^, Egypt^[31,32]^, Ghana^[33,34]^, and one each from Kenya^[35]^, Sudan^[36]^, Mali^[37]^, and Tanzania^[38]^. A total of 3730 participants were studied, and the majority of whom were in the control group (51.9%) and were female (68%). The attrition rate following the allocation of participants ranged from 0% to 44.8%. The mean age of the participants ranged from 48.8 to 55.2 years. Fourteen of the studies exclusively enrolled individuals with type 2 diabetes.

**Table 1:** Characteristics of included studies

First author (year)	Country	Diabetes type	Total sample	Female %	*I* %	Mean age	Mean time since diagnosis–years
Lamptey (2023)	Ghana	T2D	206	69.0	50.0	NR	NR
Mohamed (2021)	Egypt	T2D	100	60.0	50.0	NR	NR
Muchiri (2016)	South Africa	T2D	82	86.6	50.0	NR (I 59.4, C 58.2)	NR
Gathu (2018)	Kenya	T2D	140	44.3	50.0	48.8 (I 50.2, C 47.5)	NR
Mash (2014)	South Africa	T2D	1570	73.8	54.8	NR (I 55.8, C 56.4)	NR
Diriba (2023)	Ethiopia	T2D	76	55.3	50.0	49.4 (I 48.8, C 49.9)	5.0 (I 5.0, C 5.0)
Amendezo (2017)	Rwanda	T1D and T2D	251	69.3	60.0	50.9 (I 51.4, C 50.5)	7.0 (I 6.8, C 7.2)
Ng’ang’a (2022)	Rwanda	T2D	80	56.3	47.5	NR	NR
Badi (2024)	Sudan	T2D	364	76.4	50.0	55.2 (I 54.5, C56.0)	NR
David (2021)	Nigeria	T2D	108	68.5	50.0	50.8 (I 51.5, C 50.1)	NR
Essien (2017)	Nigeria	T1D and T2D	118	60.2	50.0	52.7 (I 52.6, C 52.8)	6.5 (I 6.9, C 6.1)
Hailu (2018)	Ethiopia	T2D	220	32.7	47.3	NR (I 55.0, C 54.0)	NR (I 10.0, C 12.0)
Abaza (2017)	Egypt	T2D	73	56.2	53.4	NR (I 51.2, C 51.8)	NR
Debussche (2018)	Mali	T2D	151	76.2	49.7	52.5 (I 53.9, C 51.1)	NR
Van Rooijen (2010)	South Africa	T2D	51	58.8	47.1	NR (I 53.2, C 54.1)	NR
Muhali (2024)	Tanzania	T1D and T2D	80	71.3	51.3	NR	NR
Asante (2020)	Ghana	T2D	60	78.3	50.0	NR (I 55.1, C 56.5)	NR (I 8.8, C 8.2)

Abbreviations: C, control group; I, intervention group; NR, not reported; T1D, type 1 diabetes; T2D, type 2 diabetes.

### Characteristics of intervention

All interventions were delivered in-person, except for two that employed a mobile health (mHealth) approach, specifically using text messages^[32]^ and mobile phone calls^[34]^. Among the in-person interventions, one utilized video content as a component of the intervention^[36]^. The interventions were delivered predominantly by healthcare professionals. Most studies used a multiple-team approach to deliver the intervention, while others were led solely by either a nurse^[26,34]^, pharmacist^[25,31,36]^, peer educator^[37]^, diabetes educator^[35]^, or a health promotor^[23]^. Eight of the interventions were organized as group session, involving 4 to 12 participants, while four were individual-based.

The number of sessions for in-person interventions ranged from 1 to 12. The mHealth interventions included 84 educational SMS^[32]^ and 16 phone follow-up calls^[34]^. The duration of each intervention session varied across studies and was as high as 6 hours in Lamptey *et al*’s study^[33]^. The participant follow-up ranged from 3 months to 12 months. Two of the studies had two-point follow-up periods of 6 and 12 months^[22]^ and 4 and 12 months^[24]^. Various domains of diabetes management were addressed, with the majority incorporating diets and exercise therapies. All studies addressed multiple domains of diabetes management in a single intervention except for 3 studies that focused solely on either diet^[22]^ or self-monitoring of blood glucose^[30,38]^. Eight interventions used various educational materials, including posters, pamphlets, handbooks, fliers, and videos. Among the included studies, seven employed various theories or models to provide a structured approach to designing interventions. Notable among the theories or models include the social cognitive theory, dual process theory, self-efficacy theory, social learning theory, and Leventhal’s common-sense theory, patient-oriented diabetic care model, health belief model, and others (Supplementary Digital Content 2. http://links.lww.com/MS9/A851, Table S1 **Supplementary Digital Content 2:** http://links.lww.com/MS9/A851).

### Quality of included studies

The result of the quality assessment (Supplementary Digital Content 1. http://links.lww.com/MS9/A850, Figure S1 **Supplementary Digital Content 1:** http://links.lww.com/MS9/A850) revealed that all the studies had either some form of bias or lacked clarity of the domains assessed. The major area of methodological limitation was the blinding of participants and personnel. Two studies mentioned the randomization of participants yet did not adequately describe the process of random sequence generation^[29,31]^. Additionally, six studies each did not provide evidence in relation to allocation concealment^[23,28,29,31,32,38]^ and blinding of outcome assessors^[22,25,33,35,37,38]^. Other forms of bias included but not limited to studies not assessing for baseline imbalance between groups of participants.

### Meta-analysis of DSME intervention

**Figure 2.**
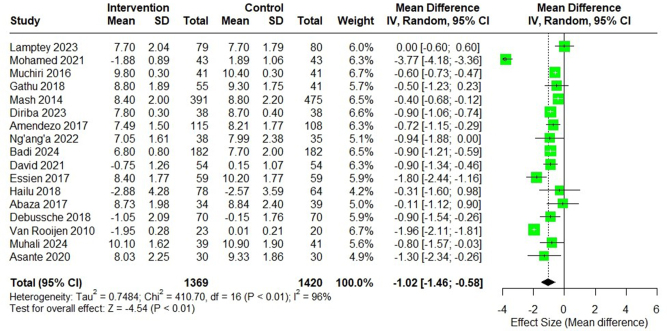
Forest plot showing the effect of DSME intervention on HbA1c.

The result of the meta-analysis (Fig. 2) revealed a significant reduction in HbA1c levels in the intervention group as compared with the usual group, with a pooled mean difference of −1.02% (95% CI −1.46 to −0.58; *I*^2^ = 96%).

### Subgroup analysis

The subgroup analysis revealed variations in the impact of DSME intervention based on the study and intervention characteristics (Table 2). However, the test of subgroup differences in the HbA1c levels was not significant except for the participant attrition rate (≥ 20%: − 0.33 vs < 20%: − 1.44; *P* <0.01). Compared to this overall estimate, a higher reduction in HbA1c was observed in DSME interventions that had multiple self-management domains, a 6-month or less follow-up period, less than a 20% participant attrition rate, and were conducted in North Africa.

**Table 2:** Subgroup analysis for DSME intervention

Variables	No. of studies	Mean HbA1c reduction	95% CI	Heterogeneity across studies	Test of subgroup difference
Format					*P* = 0.82
Group-based	8	−0.87	−1.37 to −0.37	*I*^2^ = 97%, *P* = 0.01	
Individual	4	−0.80	−1.10 to −0.50	*I*^2^ = 19%, *P* = 0.29	
DSM domain					*P* = 0.10
Multiple	14	−1.06	−1.59 to −0.54	*I*^2^ = 96%, *P* = 0.01	
One	3	−0.61	−0.74 to −0.48	*I*^2^ = 0%, *P* = 0.69	
Follow-up period					*P* = 0.56
≤ 6 months	10	−1.13	−1.82 to −0.43	*I*^2^ = 95%, *P* = 0.01	
> 6 months	7	−0.88	−1.32 to −0.44	*I*^2^ = 97%, *P* = 0.01	
Attrition rate					
< 20	9	−1.44	−2.12 to −0.75	*I*^2^ = 98%, *P* = 0.01	*P* = 0.01
≥ 20	5	−0.33	−0.56 to −0.10	*I*^2^ = 0%, *P* = 0.77	
Africa region					
Sub-Saharan	14	−0.88	−1.18 to −0.57	*I*^2^ = 95%, *P* = 0.01	*P* = 0.51
North	3	−1.62	−3.80 to 0.56	*I*^2^ = 98%, *P* = 0.01	

In accounting for sources of heterogeneity, subgroup analysis revealed that the highest heterogeneity was observed in studies with an attrition rate of <20% (*I*^2^ = 98%, *P* = 0.01), suggesting substantial variability in effect sizes within this group. Conversely, the lowest heterogeneity was found in studies that focused on a single DSM domain (*I*^2^ = 0%, *P* = 0.69) and those with an attrition rate of ≥20% (*I*^2^ = 0%, *P* = 0.77), indicating more consistent findings in these subgroups.

### Publication bias

**Figure 3.**
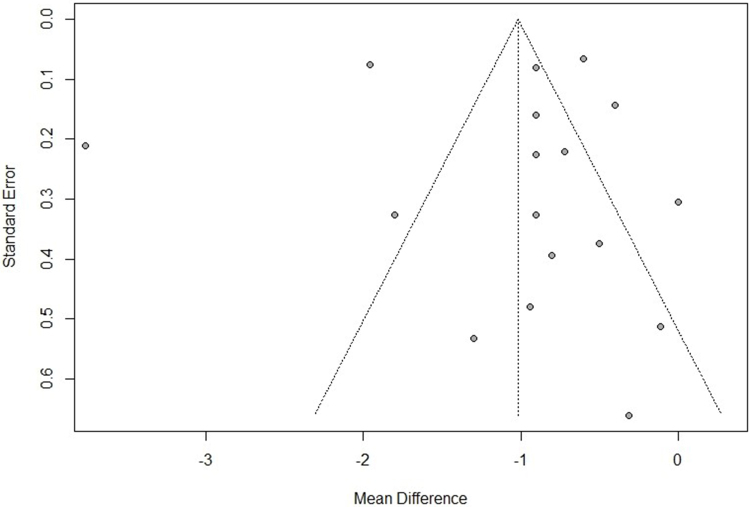
Funnel plot showing the risk of bias of included studies.

The funnel plot (Fig. 3) for the studies was relatively asymmetrical, suggesting the potential for publication bias. Further exploration using Egger’s regression-based test did not show any statistical significance of publication bias (*t* = 0.13, df = 15, *P*-value = 0.8996), suggesting the shape of the funnel plot was due to the high heterogeneity of the studies included in the analysis.

## Discussion

This systematic review and meta-analysis evaluated the effectiveness of DSME interventions on glycemic control among individuals with diabetes in Africa. By synthesizing data across 17 studies, we found that DSME interventions led to a clinically meaningful reduction in HbA1c levels (−1.02%) compared to usual care. This finding aligns with global and regional systematic reviews, which have reported HbA1c reductions ranging from 0.3% to 0.8%^[10,39,40]^.

Despite the overall positive impact, there was notable variability in intervention effectiveness across studies. This heterogeneity likely reflects differences in DSME characteristics such as duration, delivery format, and the specific self-care domains addressed^[10,41]^. For example, a previous systematic review has shown that DSME interventions lasting four months achieved a greater HbA1c reduction (0.6%) compared to those exceeding this duration (0.29%)^[10]^. Additionally, group-based DSME interventions have been found to be more effective than individual sessions^[41]^. While our subgroup analysis reflected similar trends, the test for subgroup differences was not statistically significant.

A key finding of this review is the limited use of mobile health (mHealth) technologies in DSME interventions in Africa, with only two of the included studies utilizing mHealth approaches. Given the widespread mobile phone penetration across the continent, this represents a missed opportunity for enhancing DSME accessibility. To facilitate mHealth integration, clear implementation pathways must be established^[42]^. First, culturally appropriate mobile platforms should be developed, ensuring that content aligns with literacy levels and local dialects. Second, pilot studies should assess feasibility, user engagement, and effectiveness. Finally, integrating mHealth tools into healthcare systems – such as linking interventions with primary healthcare providers for remote monitoring – will be essential for long-term sustainability. Future research should explore the long-term impact of mHealth-enhanced DSME interventions on glycemic control, adherence, and patient outcomes.

While our findings underscore the positive impact of DSME, it is essential to recognize that contextual factors may influence its effectiveness in Africa. Financial constraints frequently limit access to diabetes education programs, while healthcare systems, particularly in rural areas, often lack trained personnel and infrastructure to deliver DSME effectively^[43]^. Additionally, cultural beliefs, including the perception of diabetes as a spiritual affliction, contribute to reluctance in adopting biomedical approaches such as DSME. Addressing these challenges requires region-specific solutions that acknowledge the sociocultural and economic realities of African populations.

To support the translation of evidence into practice, healthcare policymakers should consider several targeted approaches (Table 3). Integrating traditional healthcare practitioners into DSME programs could enhance accessibility, as they are often the first point of contact in many African communities^[44]^. Training these practitioners to provide basic diabetes education and support may help bridge cultural and accessibility gaps. Additionally, cost remains a major barrier, necessitating government and health agency initiatives to subsidize diabetes education programs, glucose-monitoring devices, and essential medications^[43]^. Developing culturally adapted educational materials – incorporating local languages, traditional diets, and community health perspectives – could further improve engagement and adherence. Community health workers and peer educators should play a central role in delivering these interventions. Given the shortage of diabetes specialists, task-shifting strategies – where nurses and community health workers are trained to provide DSME – could expand intervention coverage, particularly in underserved areas.

**Table 3:** Strategies to Enhance DSME Implementation in Africa

Strategy	Implementation Approach
Integration of Traditional Healers	Train traditional healthcare practitioners to provide basic diabetes education and self-management support to improve accessibility.
Culturally Adapted Educational Materials	Develop DSME materials in local languages, incorporating traditional diets and cultural perspectives to improve engagement.
Task-Shifting Approaches	Train nurses, community health workers, and peer educators to deliver DSME interventions in underserved areas to address the shortage of diabetes specialists.
Use of mHealth Technologies	Expand DSME delivery via mobile platforms, ensuring content is culturally and linguistically appropriate. Conduct pilot studies to assess feasibility and effectiveness.
Health System Integration	Embed DSME within primary healthcare settings, ensuring routine follow-up and linking patients with healthcare providers for sustained support.

### Strength and limitations

While there are existing systematic reviews related to DSME, most included few studies from Africa^[10,45,46]^ and focused on only type 2 diabetes^[10,46]^. This present study consolidates evidence from 17 studies, offering an updated and comprehensive perspective on DSME intervention effectiveness in Africa. Our analysis highlights key gaps, notably the limited use of mHealth and culturally tailored DSME interventions, both of which represent critical areas for future research. However, several limitations warrant consideration. First, our meta-analysis revealed a high level of heterogeneity, which may limit the generalizability of the findings. Additionally, our review was limited to studies published in English, which may have excluded relevant studies published in other languages. Future systematic reviews should consider multilingual publications to mitigate this limitation.

## Conclusions

This systematic review reaffirms the effectiveness of DSME interventions in improving glycemic control among individuals with diabetes in Africa. However, addressing socioeconomic and cultural barriers, leveraging mHealth technologies, and implementing sustainable intervention models are crucial for maximizing impact. Future research should focus on the long-term sustainability of DSME interventions and the integration of digital health solutions into diabetes care frameworks across Africa. By adopting context-specific strategies, policymakers and healthcare practitioners can enhance the reach and effectiveness of DSME, ultimately improving diabetes outcomes on the continent.

## Data Availability

All data for this review can be accessed in this manuscript and its supplementary files.

## Research registration unique identifying number (UIN)

PROSPERO (Registration ID: CRD42024565320).
